# ZIF-12/Fe-Cu LDH Composite as a High Performance Electrocatalyst for Water Oxidation

**DOI:** 10.3389/fchem.2021.686968

**Published:** 2021-06-24

**Authors:** Arslan Hameed, Mariam Batool, Waheed Iqbal, Saghir Abbas, Muhammad Imran, Inayat Ali Khan, Muhammad Arif Nadeem

**Affiliations:** ^1^ Catalysis and Nanomaterials Lab 27, Department of Chemistry, Quaid-i-Azam University, Islamabad, Pakistan; ^2^ Department of Biological Sciences, National University of Medical Sciences, Rawalpindi, Pakistan; ^3^ Department of Chemistry, Faculty of Sciences, King Khalid University, Abha, Saudi Arabia; ^4^ Chemistry of Interfaces, Luleå University of Technology, Luleå, Sweden

**Keywords:** composite, co-precipitation, electrocatalysts, water oxidation, tafel analysis

## Abstract

Layered double hydroxides (LDH) are being used as electrocatalysts for oxygen evolution reactions (OERs). However, low current densities limit their practical applications. Herein, we report a facile and economic synthesis of an iron-copper based LDH integrated with a cobalt-based metal-organic framework (ZIF-12) to form LDH-ZIF-12 composite (1) through a co-precipitation method. The as-synthesized composite **1** requires a low overpotential of 337 mV to achieve a catalytic current density of 10 mA cm^−2^ with a Tafel slope of 89 mV dec^−1^. Tafel analysis further demonstrates that **1** exhibits a slope of 89 mV dec^−1^ which is much lower than the slope of 284 mV dec^−1^ for LDH and 172 mV dec^−1^ for ZIF-12. The slope value of **1** is also lower than previously reported electrocatalysts, including Ni-Co LDH (113 mV dec^−1^) and Zn-Co LDH nanosheets (101 mV dec^−1^), under similar conditions. Controlled potential electrolysis and stability test experiments show the potential application of **1** as a heterogeneous electrocatalyst for water oxidation.

## Introduction

Water is an important renewable energy source and has the potential to meet current energy crisis needs via photochemical, electrochemical, and photoelectrochemical splitting to produce oxygen and hydrogen green fuels (Conti et al., 2016; Shao et al., 2018). Oxygen evolution reaction (OER) is the most crucial reaction of water splitting. OER is considered as strenuous in contrast to HER due to sluggish kinetics (Walter et al., 2010; Man et al., 2011) since OER is a four electron process and involves simultaneous fragmentation of the O-H bond and formation of an O=O bond that needs 1.23 V vs RHE (Symes and Cronin, 2013).

Noble metals (such as Ir, Pt, and Ru) based heterogeneous and homogeneous electrocatalysts have been reported as benchmark electrocatalysts which show high activity and low overpotential values toward water oxidation. However, due to their scarcity, high cost, and instability in alkaline medium, commercial application of these precious metal catalysts is restricted (McCrory et al., 2013; Symes and Cronin, 2013; Lattach et al., 2014; Sheridan et al., 2015). Focus has now been given to the abundant and non-precious materials which can replace these benchmark electrocatalysts (Zhu et al., 2019). Diverse inorganic materials including metal oxides (Surendranath et al., 2009; Smith et al., 2013; McCrory et al., 2015; Yamada et al., 2020), amorphous materials (Zhou et al., 2013), perovskite structures (Kudo et al., 2000; Sabba et al., 2015; Jin and Bard, 2020a), hydro(oxy)oxide (Song and Hu, 2014), chalcogenides (Gao et al., 2012), olivines (Lee S. W. et al., 2012), and polyoxometalates (Stracke and Finke, 2011; Soriano-López et al., 2013; Han et al., 2014) have been explored as potential candidates for OER catalysis.

Layered double hydroxides (LDH), also known as hydrotalcite-like clays, have stacking of brucite octahedral layers. Space between the cationic layer host anions to compensate for the positive charge of the layer (Long et al., 2014). These anions are replaceable. So, these materials exhibit a specific property as an anion exchanger, which makes them highly attractive in the field of catalysis (Khan et al., 2016). In LDH class, the metal cations from transition element groups undergo redox reaction under applied potential range. Metal cations in the layer have been supposed to enhance the charge transport of the catalyst. Electron hopping mixed mechanism along the layer is believed to be a reason for charge transport, which is ascribable to the inner redox reaction between oxidized and reduced forms of metal cations (Aguilar-Vargas et al., 2013).

NiFe-LDH, (Hunter et al., 2016), CoMn-LDH (Wang J. et al., 2016), and NiCo-LDH (Yu et al., 2016) have been reported as efficient electrocatalysts for water oxidation in alkaline medium. Muller et al., presented that oxygen evolution activity of [NiFe-LDH] nanosheets is associated with Pk_a_ of the conjugate acid of interlayer anions (Hunter et al., 2016). Similarly, Sun et al., reported a three-dimensional porous film of NiFe-LDH nanoparticles as an extremely efficient and durable oxygen evolution catalyst showing low onset overpotential of 320 mV, Tafel slop of 50 mV dec^−1^, and water oxidation current density of 60 mA cm^−2^ (Yu et al., 2016).

Metal organic frameworks (MOFs) are a new class of microporous and crystalline materials (Aiyappa et al., 2019) which attained considerable attraction toward catalytic reactions due to their inherent features, including their large surface area (He et al., 2019), tunable porosity, and tailorable functionality (Corma et al., 2010; Gascon et al., 2014). For electrocatalysis, these materials are considered a promising template for the synthesis of metallic compounds and carbon-based porous materials by post calcination treatment. Active metal center and pre-functionalized organic ligands also have great electrocatalytic properties. MOFs show the characteristics of heterogenous catalysts (Wang and Wang, 2015; Wang and Wang, 2016). Undoubtedly, MOFs offer great promise as oxygen evolution reaction electrocatalysts because the accessible and tunable pores and open channels in MOFs can provide the accommodation to electrolytes, facilitate diffusion process of the reactants, and assist the transport/evolution of generated oxygen gas. Moreover, homogeneous distribution of metal cations in MOFs can serve as the catalytic active sites for OER, while ligands in frameworks would control the redox switching properties of neighboring metal cations through diversifying its coordination mode or chelating fashion (Ryu et al., 2015).

MOF-based nanomaterials have been found to be highly active for CO_2_ photoreduction (He X. et al., 2017). Recently, many MOF-based materials have been reported for OER, i.e., Bulk Ni.Co-MOF (Thangasamy et al., 2020), 2D Co-MOF nanosheets (Xu et al., 2018), 2D Ni-MOF@Fe-MOF nanosheets (Wan et al., 2017), Cobalt-based MOF ZIF-9 (Shen et al., 2017), ZIF-67 (Xia B. Y. et al., 2016), MOF-74 (Lu et al., 2017), ZIF-8 (Amiinu et al., 2017), 2D Cobalt MOF (Guan et al., 2017), and Ni@NC-800 (Xu et al., 2017). There is a great focus on fabricating MOFs for enhanced conduction and improved catalytic applications (Yu et al., 2016; Song et al., 2020). Zhou et al., reported Ni-based metal organic frameworks synthesized by using 4,4-biphenyldicarboxylic acid as ligand for high performance supercapacitor application where it exhibits higher specific capacitance, rate capability, operating current density, charge transfer resistance, high energy density, and ion diffusion impedance (Cao F. et al., 2017).

Herein, we have explored the synergistic effect between a cobalt-based zeolitic imidazolate framework (ZIF-12) and Fe-Cu-based LDH toward OER. Iron is an active metal that enhances the activity of a composite (Anantharaj et al., 2017). While copper in +2 oxidation state is a hard metal that is conductive in nature and its rigidity provides stability to the catalyst (Wang T. et al., 2018). Incorporation of iron species in LDH structures dramatically enhances OER activity. This behavior has been attributed to the Lewis acidity of Fe(III) (Li et al., 2017). However, the Fe(III) is more than a Lewis acid. These redox active ions in the LDH lattice cause a charge imbalance in M(OH)_6_ layer that is compensated for by the intercalated anions (Li Z. et al., 2015). Boettcher recommends layered structures as critically important for highly efficient water oxidation catalysis (Trotochaud et al., 2012). The main purpose of incorporation of metal-organic framework with LDH is to increase surface area and roughness factor. Here, we have chosen cobalt-based MOF because of its rich redox properties and distinctive ability to form high oxidation cobalt species during electrolysis that are critical for OER catalysis (Liang et al., 2011; Li et al., 2013; Li et al., 2016; Jin and Bard, 2020b). Due to the porous nature of MOF, the roughness factor increases. The greater the roughness factor (*R*_f_) is, the greater the activity (Xia C. et al., 2016) will be (*R*_f =_ *C*_dl_/60 mF cm^−2^). ZIF-12/FeCu-LDH composite **1**) has shown a remarkable activity with a low overpotential value, low Tafel slope, and excellent stability in alkaline conditions toward electrocatalytic OER.

## Experimental

### Synthesis of ZIF-12

**SCHEME 1:**
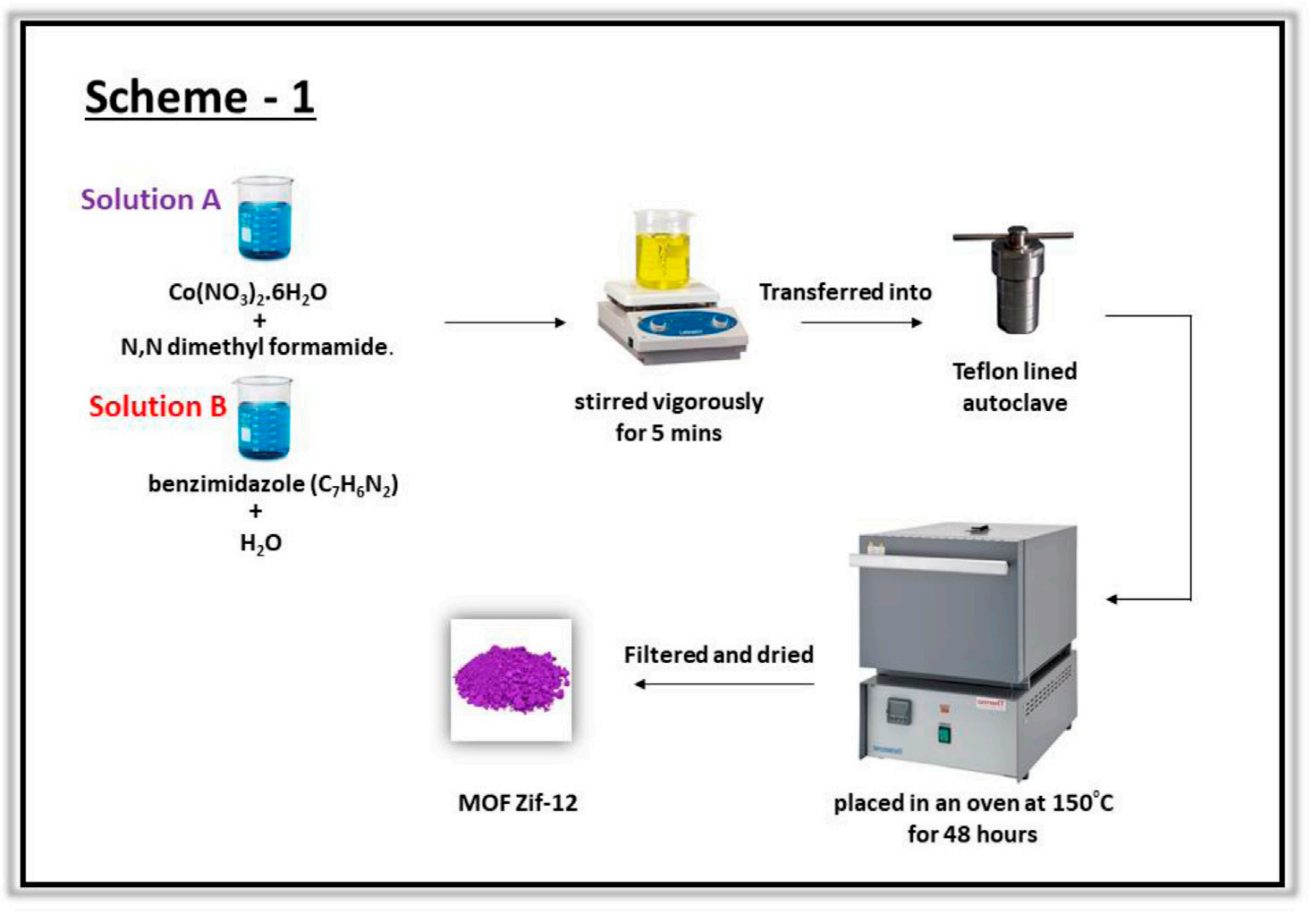
Schematic illustration of the synthesis of ZIF-12.

The cobalt imidazolate framework (ZIF-12) was synthesized using a solvothermal process as described previously (He et al., 2013). A solution of cobalt nitrate was prepared by adding 410 mg of Co(NO_3_)_2_.6H_2_O to 7 ml of *N,N*′-dimethyl formamide (DMF). Another solution was prepared by adding 720 mg of benzimidazole (C_7_H_6_N_2_) to 7 ml of distilled water. Both the solutions were mixed, stirred vigorously for 5 min, and transferred into a 20 ml Teflon-lined autoclave and placed in an oven at 150°C for 48 h. After reaction completion the autoclave was cooled to room temperature and the product was collected after filtration and washed with DMF as shown in (Scheme 1).

### Synthesis of ZIF-12/Fe-Cu LDH Composite (1)

**SCHEME 2:**
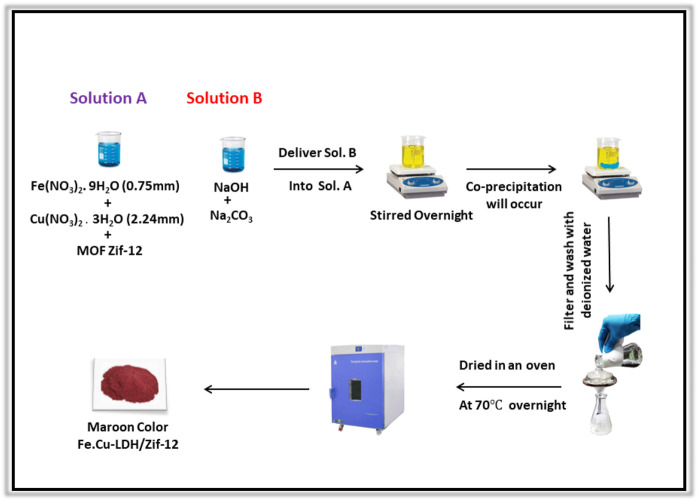
Schematic illustration of the synthesis of composite **1.**

Co-precipitation method was used for the synthesis of composite **1**. Initially, 354 mg of ZIF-12 was suspended in a solution of Fe(NO_3_)_2_.9H_2_O (171 mg) and Cu(NO_3_)_2_.2H_2_O (362 mg) to make a stoichiometric ratio of 2:1:3. A solution containing both (OH^−^) and interlayer anion CO_3_^2−^ was added dropwise through burette. The mixture was stirred overnight, and the product precipitates were collected, washed with deionized water, and dried under vacuum as shown in (Scheme 2).

### Instrumental Characterization

Details of instrumental characterizations are available in the supplementary information.

### Fabrication of Working Electrode

For the fabrication of a working electrode, an ink of the desired catalyst was prepared by adding 5 mg of **1**–2 ml of analytical grade ethanol, with 20 µl Nafion as a binder, and then sonicated for 3 h. After sonication, ink was coated on the surface of a fluorine-doped tin oxide (FTO) coated glass slide by using the drop casting method. The coated FTO electrode was dried in an oven at 70°C overnight.

## Results and Discussion

### Characterization

**FIGURE 1:**
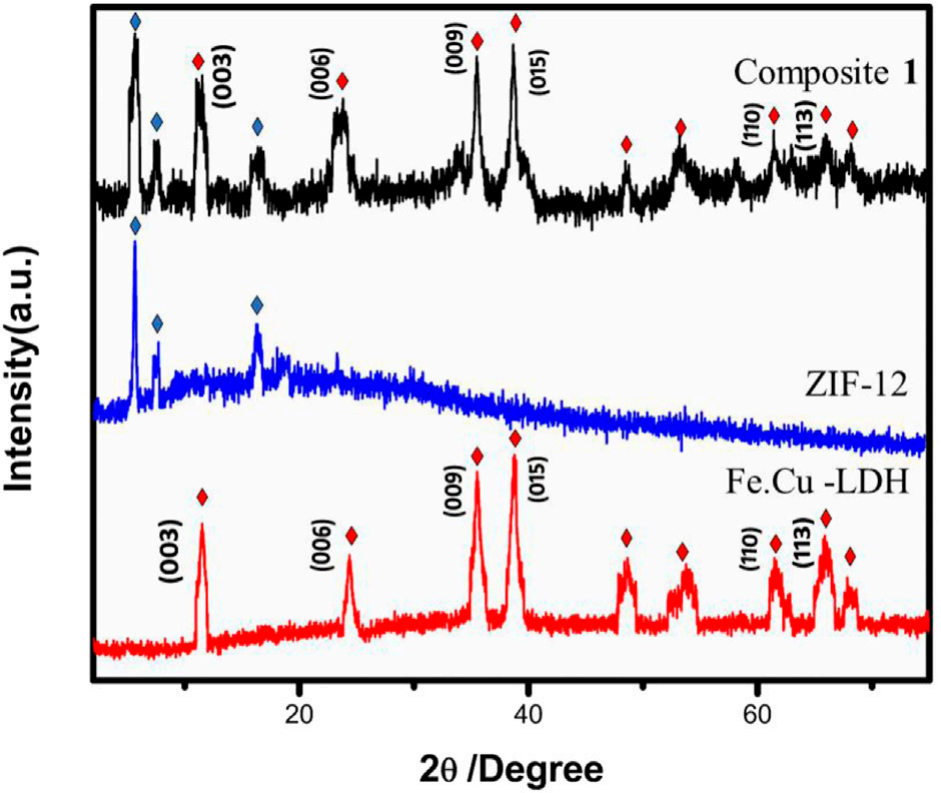
PXRD patterns of LDH, ZIF-12, and composite **1**.

The structural investigation was carried out via powder X-ray diffraction (PXRD) analysis. The PXRD patterns of FeCu-LDH, ZIF-12 and composite **1** are shown in Figure 1. The PXRD pattern of the composite have all the characteristic peaks of Fe.Cu-LDH and ZIF-12, which reveals that the incorporation of Fe.Cu-LDH in ZIF-12 does not change the framework morphology. The characteristic peaks relevant to LDH are at 2θ = 12°, 37°, and 39° and to ZIF-12 at 2θ = 4°, 6°, and 15°, which are shown in the PXRD pattern of composite **1.** In x-ray diffractogram (003), (006), and (009) basal plane peaks appear, corresponding to the stacking of the lamellae, characteristic of the LDH structure (Cao A. et al., 2017; Wang et al., 2020; de Melo Costa-Serge et al., 2021) (Figure 1). Electrostatic force of interactions are expected to exist at the interface of both Fe.Cu-LDH and ZIF-12. This is due to the presence of hydroxyl ions (OH^−^) in LDH and cationic metal sites in ZIF-12. From the PXRD pattern, it is clear that there is no major shift in the peak positions of ZIF-12 which represent that ZIF-12 retained its structural integrity in the composite **1**.

**FIGURE 2:**
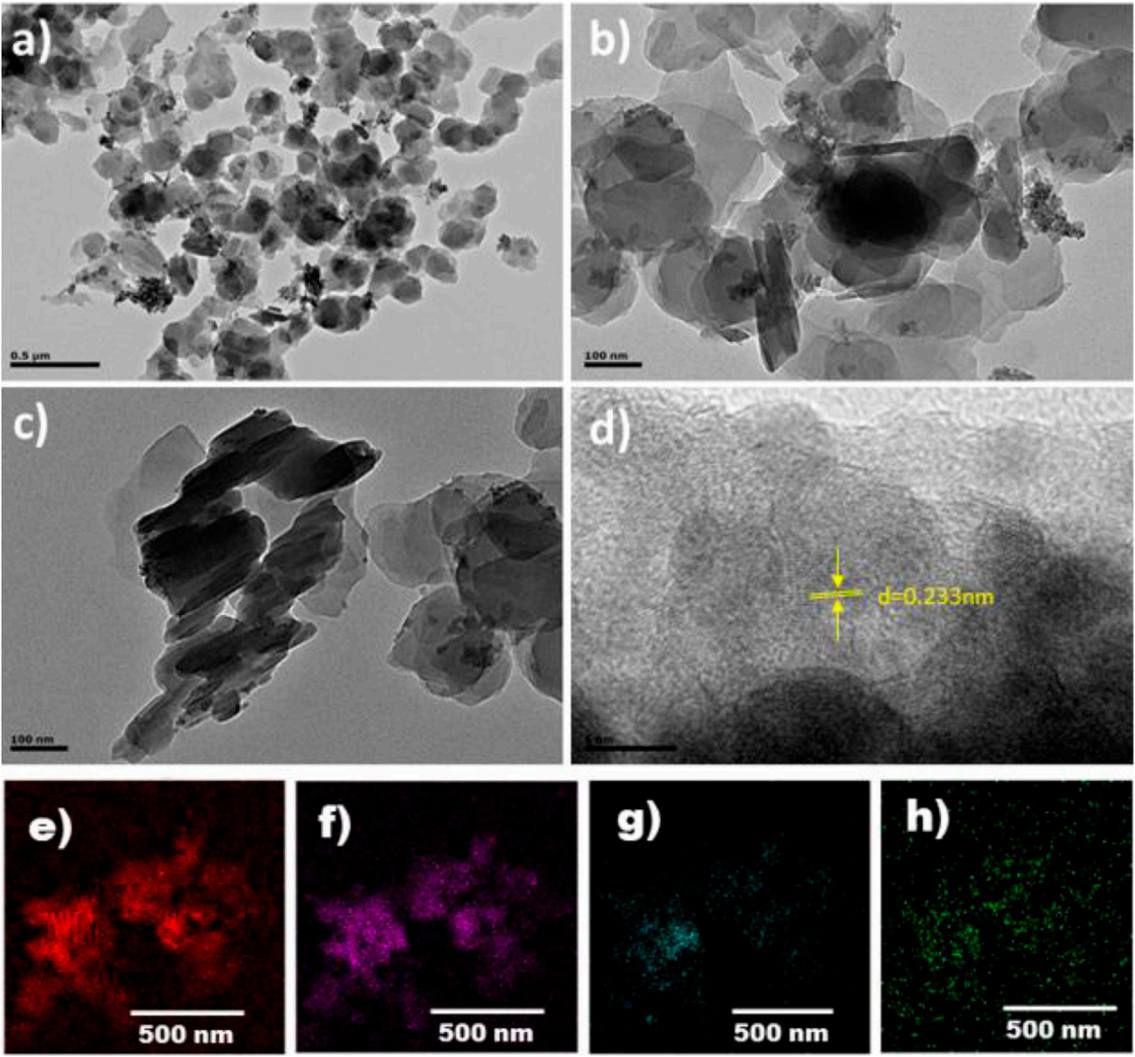
TEM images of composite **1 (A–D)**. Elemental mapping with element distributions: carbon **(E)**, copper **(F)**, cobalt **(G)**, and iron **(H)**.

Transmission electron microscopy (TEM) was carried out to observe the morphology and particle size of the composite **1** ingredients; the images are shown in Figure 2A,B. The LDH particles mostly exist in nanosheet structures. There are also some particles of LDH where many LDH layers overlap to form a multilayer structure (Figure 2C). Figure 2D shows the high resolution TEM image where *d*-spacing of 0.233 nm corresponds to (015) crystal planes of LDH (Liu et al., 2019). This plane corresponds to 2θ = 39 in PXRD pattern. Lattice fringes in the TEM images confirm the presence of layer double hydroxide (LDH) in the MOF linings (Li et al., 2010; Valdez et al., 2015; Wang S. et al., 2016). The element mapping further reveals the presence of Fe, Cu, Co, and C elements distributed uniformly (Figures 2E–H).

**FIGURE 3:**
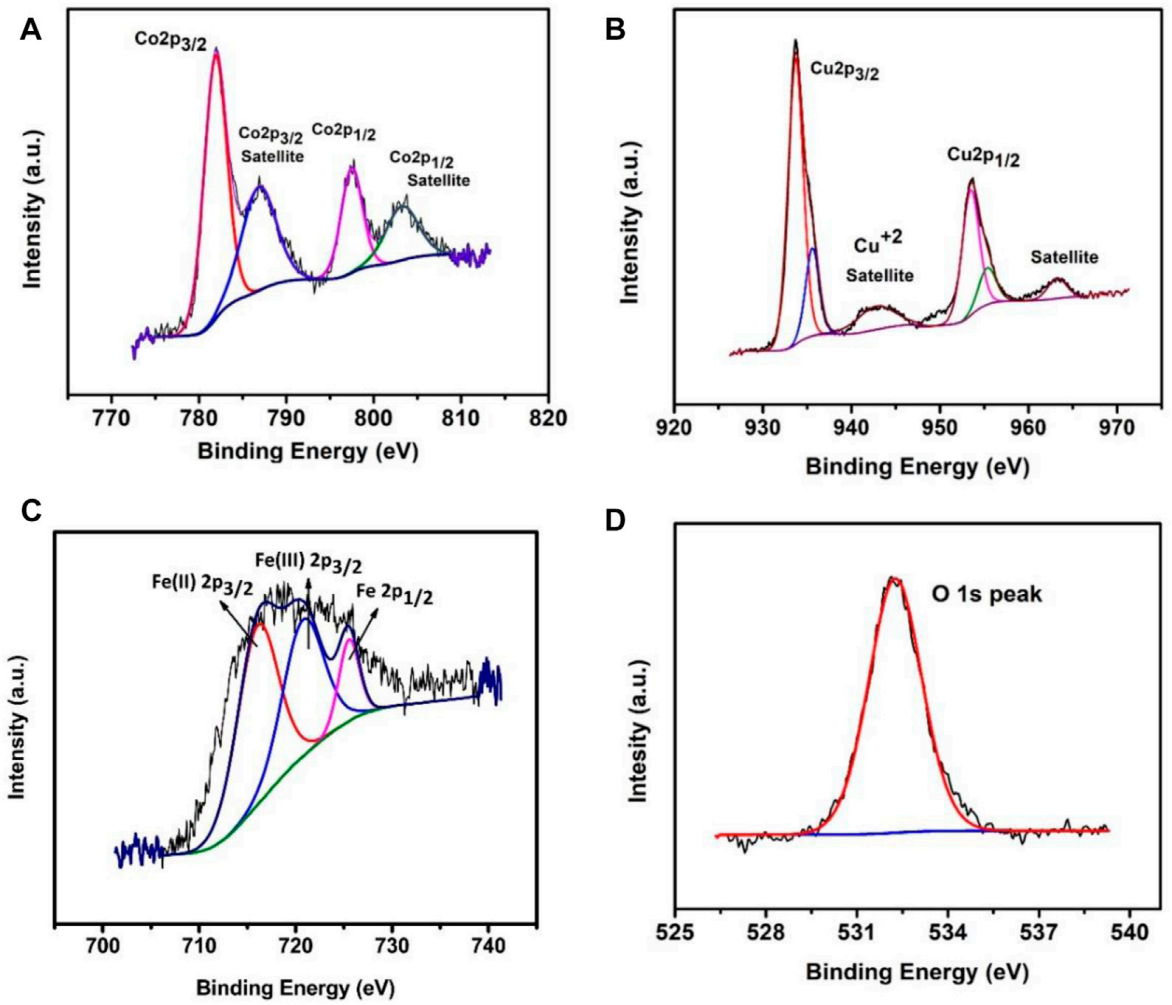
The X-ray photoelectron spectroscopy (XPS) spectra: Co 2p **(A)**, Cu 2p **(B)**, Fe 2p **(C)**, and O 1s **(D)**.

The X-ray photoelectron spectroscopy (XPS) studies of catalytic samples (Figure 3) confirms the existence of Fe, Cu, Co, C, and O elements. As shown in Figure 3A, two peaks at 780.7 and 795 eV are assigned to the binding energy of Co 2*p*_3/2_ and Co 2*p*_1/2_, respectively (Jiang et al., 2011; Yao et al., 2011), with two satellite peaks which are located at 786 and 803 eV and can be assigned to Co 2*p*_3/2_ satellite and Co 2*p*_1/2_ satellite, respectively (Yao et al., 2011; Zhang et al., 2016; Hada et al., 2001). The value of Co 2*p*_3/2_ is distant from the value of Co^0^ (i.e., 777.6 ± 0.7) but close to the value of Co^2+^ (i.e., 779.8 ± 0.8) which shows that cobalt is in +2 oxidation state. Figure 3B shows two peaks at 934 and 954 eV that are assigned to the binding energy of Cu 2*p*_3/2_ and Cu2*p*_1/2_ respectively with the satellite peak located at 943 eV that can be assigned to Cu 2*p*_3/2_ satellite, which indicates +2 oxidation state of copper. The chemical oxidation state of iron in Fe.Cu-LDH/ZIF-12 was investigated by XPS spectra. The peaks at 716.07 and 725.52 eV are attributed to 2p3/2 and 2p1/2 spin state of Fe(III) for LDH lamellar structure (Rajeshkhanna et al., 2018; Zhu et al., 2018) as shown in Figure 3C. At the same time, a satellite peak located at 720.76 eV also corresponds to the Fe(III) oxidation state. While Figure 3D shows the XPS result of oxygen, where a peak appears at 532.6 eV which corresponds to the metal hydroxides (Yu et al., 2013). XPS was also used to examine the composition of the catalyst, before and after catalytic activity. Supplementary Figure S3 shows the spectra observed for the C 1*s*, N 1*s*, O 1*s*, Fe 2*p*, Cu 2*p,* and Co. 2*p* regions for pristine and post-catalytic samples.

The chemical composition of as obtained composite **1** was further determined by EDX (Supplementary Figure S4 and Supplementary Table S1). Thermogravimetric analysis (TGA) curve shows that thermal decomposition occurs in two steps (Supplementary Figure S2). In the first step, 14% weight loss has been observed within the range of 100–250°C due to the removal of adsorbed water molecules and other ions. In the second step, 52% weight loss has been observed within the range of 250–350°C due to the removal of benzimidazole that follows the formation of metal oxides.

### Oxygen Evolution Reaction Studies

**FIGURE 4:**
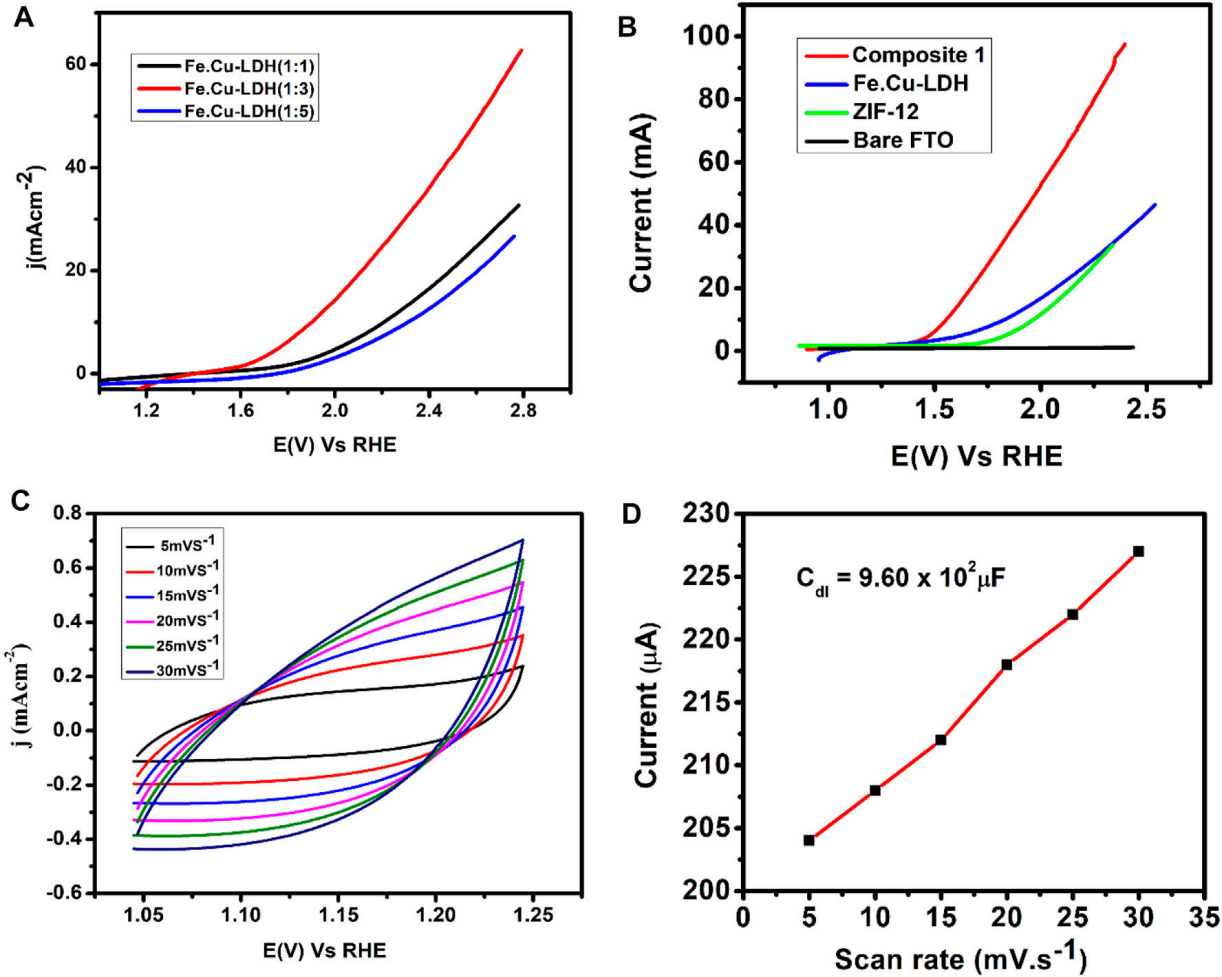
**(A)** LSV curves of FeCu-LDH with different ratios **(B)** Linear sweep voltammograms of LDH, ZIF-12, and **1**, **(C)** Cyclic voltammogram of non-faradaic region of Composite **1** at different scan rates, and **(D)** plot of anodic current vs scan rates of **1** coated electrode KOH solution (1 mol L^−1^) as an electrolyte.

Electrochemical OER performance of **1** was tested using a three-electrode system in KOH solution (1 mol L^−1^). Figure 4A shows OER performance of FeCu-LDH with a different ratio, (1:3) found to be an optimum composition ratio toward OER activity. In Fe.Cu-LDH, the active sites are iron metal (Burke et al., 2015) while copper provides conductivity; when we increase the ratio of copper, the conductivity increases to some extent, and OER activity also increases. Further increases in copper ratio decreases OER activity because copper metal replaces most of the active site in (1:5), which causes a decrease in OER performance (Burke et al., 2015). A linear sweep voltammogram of a bare FTO electrode indicated that the bare FTO has almost negligible activity toward OER, generating an insignificant amount of current (Figure 4B). Hence, it can be concluded that the current density obtained is due to oxygen evolution reaction. An LSV curve of pure iron-copper LDH and ZIF-12 coated on FTO showed that both catalysts exhibited good activity toward water oxidation and produced current densities of 46 mA cm^−2^ and 30 mA cm^−2^, respectively.

It is important to note that the activity of iron-copper layer double hydroxide was significantly enhanced by the incorporation of ZIF-12. LSV curve of composite **1** exhibited much improved OER performance by producing a current density of 96 mA cm^−2^. LSV results also demonstrate a significant shift in the onset potential. Composite **1** indicated water oxidation peak at an onset potential of 1.4 V vs RHE while it was observed at 1.71 and 1.69 V vs RHE for LDH and ZIF-12, respectively. These results revealed that the incorporation of ZIF-12 into FeCu-LDH has increased the efficiency of the catalyst.

The ideal overpotential for OER is 10 mA cm^−2^, which is a conventional value to estimation current density (*j*) applicable to solar fuel synthesis with 12% solar to H_2_ activity (Song and Hu, 2014). The composite **1** showed fast kinetics for OER by producing a current density of 10 mA cm^−2^ at an overpotential of 337 mV (Figure 4B). The overpotential required for **1** is comparable to NiFe-HT (more than 0.32 V) and NiFe-A (0.34 V) (Lu et al., 2014) but is much lower than those required for LDH only (470 mV), ZIF-12 (510 mV), CoP/rGO hybrids (340 mV) (Jiao et al., 2016), carbon fiber paper@FeP (350 mV) (Xiong et al., 2016), CoP hollow polyhedron (400 mV) (Liu and Li, 2016), and Ni–Co LDH nanoboxes (420 mV) (He et al., 2017b). In order to further investigate and compare the catalytic efficiency, **1** showed a mass activity of 18.86 A g^−1^ at an overpotential of 0.34 V which is comparable to the mass activity reported for the benchmark Ir/C (9 A g^−1^, 0.38 V, 0.1 M KOH) electrocatalyst (Lee Y. et al., 2012).

Furthermore, another criterion to investigate and compare the catalytic efficiency of different electrocatalysts under similar experimental conditions is the turnover frequency (TOF) calculation of the catalyst. Composite **1** exhibited a TOF of 0.01 s^−1^ at an overpotential of 337 mV.

The electrochemical double-layer capacitance (*C*_dl_) of catalytic sites is another important parameter to evaluate the catalytic efficiency of the designed materials and it is associated with the electrochemical active surface area (ECSA) [4, 43–46]. *C*_dl_ value can be determined by adopting two pathways: 1) By measuring the charging currents or capacitive currents obtained from the scan rate dependent cyclic voltammograms in the non-Faradaic capacitive current region (Ibrahim et al., 2019), 2) Employing electrochemical impedance spectroscopy (EIS) for the estimation of the frequency reliant impedance of the electrocatalytic system (Zhuang et al., 2012; Brug et al., 1984; Huang et al., 2007). In this regard, a potential sweep window was selected in the non-faradic capacitive current region of the LSV scan by visual estimation of LSV data considering that all the current within that potential range is produced only due to the electrical double-layer charging. Under the chosen potential range, LS voltammograms were run at variable scan rates (5–30 mV s^−1^) (Figure 4C). The capacitive current was calculated by spotting a single potential value (1.15 V vs RHE) somewhere in the non-Faradaic capacitive potential window. The plot of anodic current vs. scan rates in the range from 5 to 30 mV s^−1^ gave a straight line with a slope equivalent to *C*_dl_ (Figure 4D) (Zou et al., 2013). The measured double layer capacitance from this analysis is 0.96 mF cm^−2^, which is much less than NiCoP/C nanoboxes (146 mF cm^−2^), Ni–Co LDH nanoboxes (9.15 mF cm^−2^), and NiCoP nanoboxes (28.93 mF cm^−2^) (He et al., 2017a). The obtained *C*_dl_ value indicated that the current density obtained in the catalytic region arises only because of the faradaic processes, as the measured charging currents values are insignificant.

**FIGURE 5:**
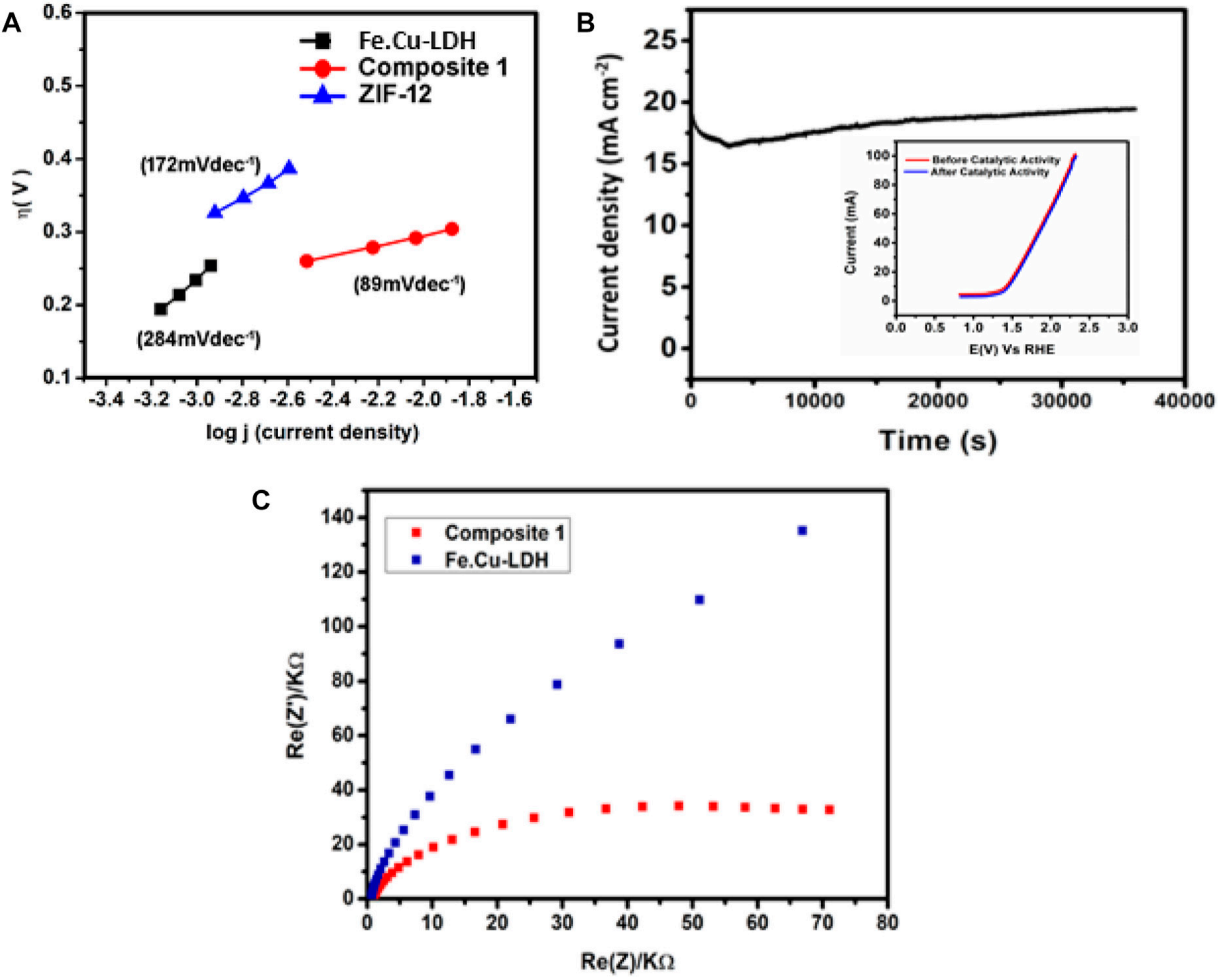
**(A)** Tafel slops of Fe.Cu-LDH, ZIF-12 and **1**, **(B)** controlled-potential electrolysis (CPE) of **1** in basic conditions and (inset) LS voltammograms of pristine and post-catalysis of **1** modified FTO electrodes, and **(C)** electrochemical impedance spectra of non-faradaic region of **1** coated electrode recorded in KOH solution (1 mol L^−1^) as an electrolyte.

In Figure 5A Tafel analysis demonstrated that **1** exhibits a slope of 89 mV dec^−1^ which is much lower than the Tafel slope values obtained for LDH (284 mV dec^−1^) or ZIF-12 (172 mV dec^−1^) in the current study. This value is also lower than previously reported electrocatalysts, i.e., Ni-Co LDH (113 mV dec^−1^) (Yu et al., 2016) and ZnCo LDH nanosheeets (101 mV dec^−1^) (Aiyappa et al., 2019), under the same conditions.

An advantageous parameter to elucidate the oxygen production performance of a catalyst is to determine the Faradaic efficiency (FE). FE is obtained by comparing the experimental and theoretical yield of evolved oxygen during a controlled-potential electrolysis (CPE) (Shah et al., 2018). In order to investigate the FE of the catalyst, a CPE experiment was performed at a constant potential of 1.45 V vs RHE for 3,000 s using similar electrochemical reaction conditions. In this regard, an oxygen probe of the dissolved oxygen (DO) meter was inserted into an air-tight anodic compartment purged with N_2_ gas for ten minutes, before the experiment, and the concentration of DO was monitored for an hour to establish a baseline. The charge accumulated during the electrochemical reaction was used to calculate the theoretical yield of O_2_ applying Faraday’s law for a four-electron process. The proximity between the amounts of DO detected during CPE and the theoretically measured oxygen evolution yield concluded FE of 77% and ruled out the possibility of a side reaction. The FE was calculated from theoretical and actual yield, which is about 77% (Supplementary Figure S1). A possible reason for composite 77% FE is probably the metals oxidation’s current contribution along with water electrolysis.

In order to assess the long-term stability and robustness of composite **1,** a controlled-potential electrolysis (CPE) measurement was carried out for 10 h using chronoamperometry at 1.65 V vs RHE under constant experimental conditions as presented in Figure 5B. The CPE test indicated that the catalyst produced an excellent current density of 18.3 mA cm^−2^ that remained constant until the last minute of CPE. A vital test to evaluate the robustness of **1** after the catalytic activity can be performed by performing LSV measurements of both pristine and post-catalysis samples, witnessing the onset potential for OER and recording the maximum *j* values. It is clear in the inset of Figure 5B that the LSV of **1** coated modified electrode (before and after catalytic activities) demonstrated insignificant change in the onset potential and maximum current density values which confirmed that **1** retained its structural integrity throughout the catalytic phenomenon. Noticing the CPE results, it can be inferred that **1** has strong potential to be used as a robust and efficient OER electrocatalyst. Electrochemical impedance spectroscopy has also been done to provide more insight into electrocatalytic activity. The frequency range for EIS was between 0.1 Hz and 100 kHz for both FeCu-LDH and composite **1**. A Nyquist plot of real and imaginary components of EIS in Figure 5C clearly shows that composite **1** has small arc or small charge transfer resistance as compared to FeCu-LDH, which reveals fast OER kinetics in composite **1.** The most probable mechanistic pathway for OER at electrified anode is as follows:

[composite 1]∗+ OH- → [composite 1]-HO∗ + e- (1)

[composite 1]-HO∗ + OH- → [composite 1]-O∗ + H2O (1) + e- (2)

[composite 1]-O∗ + OH- → [composite 1]-OOH∗ + e- (3)

[composite 1]-OOH∗ + OH- → [composite 1]∗ + O2 + H2O + e- (4)

The surface active site of composite **1** electrocatalyst was initiated by hydroxyl specie (OH-) from water and the removal of an electron to form composite **1**-OH, which further reacts with another OH- to form composite **1**-O- specie (Oxo-specie). This oxo-species combines with OH- to form hydro-peroxide as an intermediate composite **1**-OOH. Finally, OH- species reacts with composite **1**-OOH intermediate in step (III) to give O_2_ molecules in step (IV) of the mechanistic pathway of OER. This is in good agreement with the literature reports (Jiao et al., 2015; Suen et al., 2017; Lee et al., 2018; Sapner et al., 2020) and is summarized in Eqs. 1–4. Table 1 shows the comparative analysis of different reported benchmark electrocatalysts with composite **1.**

**TABLE 1:** Comparative analysis of different reported benchmark electrocatalysts with composite **1.**

Catalyst	Overpotentials (mV) at 10 mA cm^−2^	References
IrO_2_	411	Ma et al. (2015)
NiFe_2_O_4_	440	Li M. et al. (2015
MnO_x_	460	Huynh et al. (2014)
Co..Ni-LDHs/CP	367	Liang et al. (2015)
Co-BPDC	428	Zha et al. (2020)
Ni.Co-LDH	367	Wang et al. (2017)
Co./MIL-101	570	He et al. (2015)
Ni.MN-LDH@MWCNTs	350	Jia et al. (2016)
Fe_2_Ni-BPTC/CC	365	Wang X. L. et al. (2018)
UTSA-16	408	Jiang et al. (2017)
Fe.Co-ONP	400	Zhuang et al. (2017)
Co-OBA/C	590	Fan et al. (2017)
Fe_2_O_3_/CNT	410	Bandal et al. (2016)
FeCoNi alloy	400	Saha and Ganguli, (2017)
Fe.Cu-LDH	470	This work
Fe.Cu-LDH/ZIF-12	337	This work

## Conclusion

This work represents the integration of nonprecious metal-based LDH with ZIF-12 which provides structural and compositional advantages and could have fruitful applications in the hydrogen economy. LDH/ZIF-12 composite (**1**) has been synthesized through the co-precipitation method. Composite **1** showed enhanced OER performance as compared to individual components, i.e., iron-copper layer double hydroxide and ZIF-12. Chronoamperometric studies including controlled-potential electrolysis show that **one** offers a higher current density, requires low overpotentials, and has high mass activity, faradaic efficiency, and stable catalytic response for a longer period (ca. 10 h). Hence, it can be concluded that **1**, having excellent water oxidation performance, can be introduced as an efficient and stable electrocatalyst with magnificent commercial importance.

## Data Availability

The original contributions presented in the study are included in the article/Supplementary Material, further inquiries can be directed to the corresponding authors.
