# Supplementary material for: ZIF-12/Fe-Cu LDH Composite as a High Performance Electrocatalyst for Water Oxidation
Source: Front Chem. 2021 Jun 24;9:686968. doi: 10.3389/fchem.2021.686968 (PMC8264502; doi:10.3389/fchem.2021.686968)
Supplement: Supplementary file 1 [file DataSheet1.PDF]

Supporting Information

ZIF-12/Fe-Cu LDH Composite as a High Performance Electrocatalyst for Water Oxidation

Arslan Hameed[1], Mariam Batool[1], Waheed Iqbal[1], Saghir Abbas[1,2], Muhammad Imran[3], Inayat Ali Khan[4]*, and Muhammad Arif Nadeem[1]*

[1]*Catalysis and Nanomaterials Lab 27, Department of Chemistry, Quaid-i-Azam University, Islamabad 45320, Pakistan*

[2]*Department of Biological Sciences, National University of Medical Sciences, 46000, Rawalpindi, Pakistan*

[3]*Department of Chemistry, Faculty of Sciences, King Khalid University, Abha, Saudi Arabia*

[4]*Chemistry of Interfaces, Luleå University of Technology, SE-971 87 Luleå, Sweden*

*Contact details:

Email: manadeem@qau.edu.pk, Phone: +92-51-9064-2062 (M.A. Nadeem)

Email: inayat.khan@ltu.se, Phone: +46920491738 (I.A. Khan)

EXPERIMENTAL

Instrumental Characterization

PANalytical (X'Pert PRO 3040/60) X–ray diffractometer with Cu K_α (λ = 1.544206 Å) radiation generated at 40 kV and 15 mA was used for powder X-ray (PXRD) analysis at scan speed of 0.02 deg s^{-1}. Kratos AXIS Ultra DLD instrument with vacuum better than 2×10^{-9} mbar was used for XPS analysis. Radiation source of Al K_α (energy hν = 1486.68 eV), power of 164 W (15.2 kV and 10.8 mA) and spot size of 500 μm were used during measurements. The emitted photons was at 90° to the detector and adventitious carbon C 1*s* (284.5 eV) was used as reference and peaks in the spectrum were shifted accordingly. CasaXPS software was used for spectrum fitting with Shirley/Linear type background correction and asymmetric Gaussian/Lorentzians (0-30% Lorentzian character) was adopted. JEOL-JEM 2010F FE-TEM field-emission transmission electron microscope at operating voltage of 200 kV was used for TEM analysis. Perkin Elmer 8000 TGA machine was used for the thermogravimetric analysis (TGA) with about 2 to 4 mg of each sample from 303 to 873 K at 283 K min^{-1} under nitrogen.

Electrochemical measurements

All the electrochemical experiment performed with potentiostat (Biologic SP300 & Gamry Instruments, Interface 5000E) at room temperature by using three electrode system in KOH solution (1 mol L^{-1}, pH 13.7). Prior to each electrochemical experimental, the electrolyte solution was degassed with high purity N_2 gas for at least 30 mins. The as prepared Fe-Cu-LDH/ZIF-12 deposited on FTO with a geometric area of 1×1 cm^2 was directly used as working electrode (WE), Ag/AgCl as reference (RE), Pt-wire as counter electrode (CE). All the potentials measured through Ag/AgCl reference electrode were converted into Reversible hydrogen electrode (RHE) using Nernst Equation 1 which is as under:

$$E_{RHE} = E_{Ag/AgCl} + 0.0592\ (PH) + 0.197 \qquad (1)$$

Linear sweep voltammetry (LSV) was carried out in the potential range from 0 to 1.5 V vs Ag/AgCl. LSV scans were recorded at different scan rates from 10 to 400 mV s^{-1} with the difference of 10 mV s^{-1}. Chronoamperometry was performed in the range from 0.6 to 1.35 V with the difference of 0.02 V for 10 mins. Controlled potential electrolysis (CPE) was performed at fixed potential of 1.2 V vs Ag/AgCl for 36000 s to check the stability of catalyst.

Determination of Tafel Slope

Water oxidation kinetics was studied by Tafel plot measurements. Chronoamperometry estimation was performed to monitor the current density for O_2 evolution, "j" as a component of the overpotential, "η" in 1 mol L^{-1} KOH solution as an electrolyte of pH 13.7. Tafel slope is calculated from Tafel plot which is 89 mV dec^{-1}. The catalytic current density of 10 mA cm^{-2} was observed at 337 mV, above the thermodynamic potential of the OER showed up in.

Tafel slope was calculated by overpotential (η) and current density (j) by using Tafel Equation 2.

η = b log j + a ……………… (2)

where 'b' is the Tafel slope and 'a' is the constant.

Turnover Frequency Calculation

By using the surface coverage value obtained from the mass difference of the coated and bare FTO electrode, a minimum TOF value was calculated by following the Equation 3 given below:

TOF = Q/4t Γ …………… (3)

where Q = integrated charge through the modified FTO electrode (C cm^{-2}), Γ = Surface concentration (mol cm^{-3}) , t = time, and 4 is equal to number of electrons required for oxidation of 1 mole of O_2.[1]

Determination of Electrochemical Double-Layer Capacitance (C_{dl})

For electrochemical double-layer capacitance (C_{dl}) determinations, the scan rate dependent CV measurements were carried out between 50 to 500 mV s^{-1} over a small window where no Faradaic current was observed. The electrochemical double layer capacitance (C_{dl}) was extracted from $i_c = \nu \times C_{dl}$, where ν is the scan rate and i_c is the current density for the specific curve at 0.3 V.

Faradaic efficiency

It is the efficiency with which charge is transmitted in the system to increase the rate of catalytic reaction. It is calculated by following Equation 4:

O_2 = Q/4F …………….. (4)

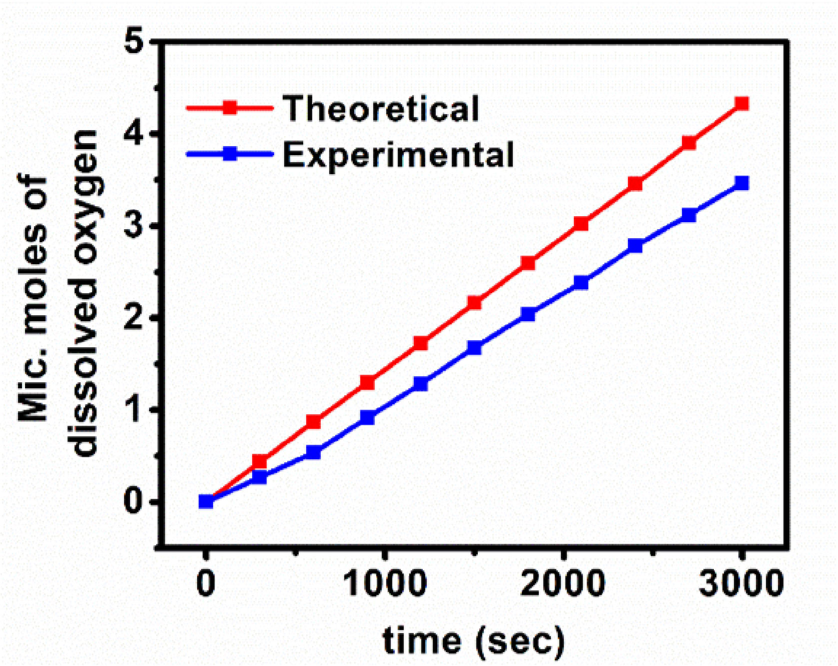

Figure S1. Oxygen production performance of the composite in reference to theoretical standards (Faradaic efficiency curve).

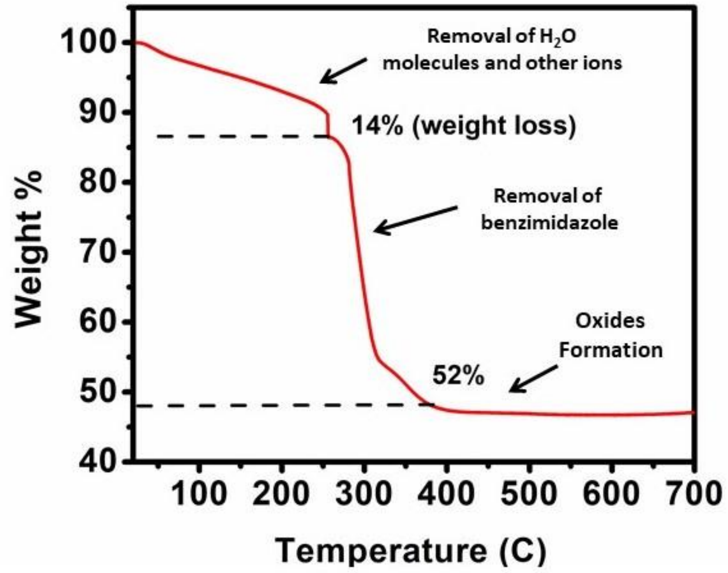

Figure S2: Thermogravimetric analysis curve of composite **1**.

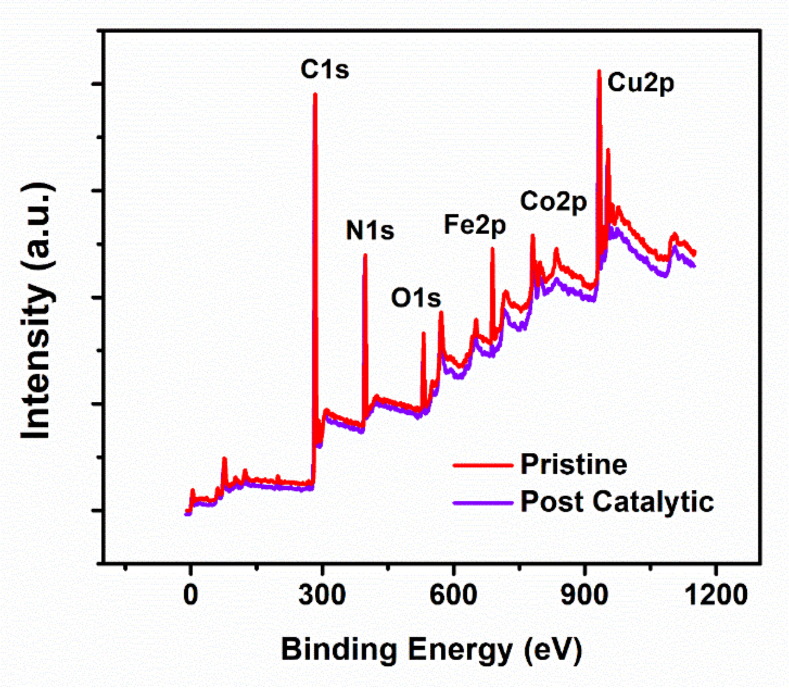

Figure S3. XPS of pristine and post catalytic composite **1**.

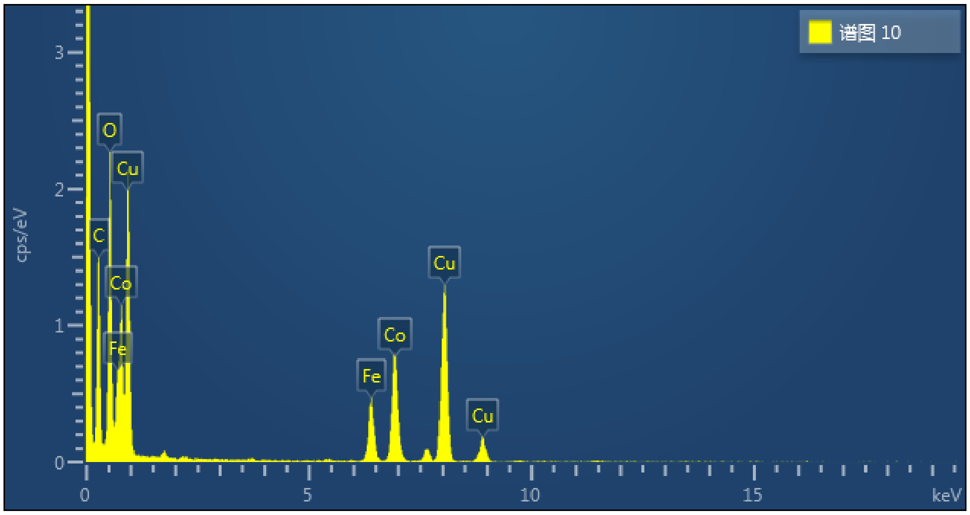

Figure S4. EDX spectrum of composite **1.**

Table S1. Energy dispersive X-ray spectroscopic data for composite **1.**

Element	The line type	The k-factor	The k-factor type	Absorption correction	wt%	wt% Sigma
C	K-Line system	3.115	theory	1.00	30.32	0.62
O	K-Line system	1.455	theory	1.00	20.81	0.43
Fe	K-Line system	1.214	theory	1.00	7.31	0.23
Co	K-Line system	1.286	theory	1.00	14.47	0.32
Cu	K-Line system	1.421	theory	1.00	27.08	0.43
Total:					100.00	

REFERENCES

1. Bard, A. J.; Faulkner, L. R., Fundamentals and applications. *Electrochemical Methods* 2001, *2* (482), 580-632.
